# Awareness of Epistaxis and Its First Aid Management Among Teachers Working in Schools of Al-Baha Region, Saudi Arabia

**DOI:** 10.7759/cureus.45670

**Published:** 2023-09-21

**Authors:** Samer A Alzahrani, Salman Alanazi, Mohammed M Alzahrani, Reema Aldhalaan, Meshari A Alghamdi, Lama F Alghamdi

**Affiliations:** 1 Family Medicine, Imam Mohammad Ibn Saud Islamic University, Riyadh, SAU; 2 Medicine, Imam Mohammad Ibn Saud Islamic University, Riyadh, SAU; 3 Otolaryngology, Al-Baha University, Faculty of Medicine, Al-Baha, SAU; 4 Medicine, Alfaisal University, Riyadh, SAU; 5 Medicine, Al-Baha University, Faculty of Medicine, Al-Baha, SAU

**Keywords:** first aid management, al-baha region, schools, epistaxis in school, teacher’s awareness, kingdom of saudi arabia (ksa)

## Abstract

Background

Epistaxis (nosebleed) is a frequent emergency presentation in the emergency department (ED). Generally, any harm to the nasal mucosa lining can lead the nose to bleed. The etiologies of epistaxis are widely classified as environmental, local, systemic, and medication-related causes. The initial management for epistaxis is first aid. First aid by applying pressure on the nostrils is essential to stop the bleeding and minimize discomfort. This study intends to evaluate teachers’ awareness regarding epistaxis first aid management inside schools in Al-Baha region, Saudi Arabia.

Methodology

A cross-sectional study was conducted among a target of 439 teachers regarding epistaxis first aid management inside schools in Al-Baha region, Saudi Arabia. Data were collected using a structured questionnaire and analyzed using descriptive statistics and a chi-square test.

Results

Of the teachers, 50.7% had received training regarding epistaxis, while 49.3% had never been trained. Also, 73.3% considered that applying nasal compression may help stop bleeding, while the rest were completely unaware. On further investigation, tilting the head forward would be done by 56.27% of the teachers, while 40.73% said that the head should be tilted back. Of the teachers, 53.3% would go to the emergency if bleeding continued for more than 10 minutes. No association between age, gender, and working area, and training received regarding the management of epistaxis was found (p>0.05), but teachers from a scientific background, as compared to those from a literature background, were able to answer better regarding the management of epistaxis (p<0.05).

Conclusion

The study highlights knowledge gaps regarding epistaxis first aid management inside schools in Al-Baha region, Saudi Arabia. The research highlights the need for focused training programs and awareness efforts to enhance teachers’ knowledge and first aid practices. Addressing misknowledge and mispractices, enhancing the practices and attitudes of healthcare providers toward appropriate feeding practices, and promoting a supportive and safe environment could all contribute to the improvement of quality of life and health among the population of Saudi Arabia.

## Introduction

Epistaxis, also known as nosebleed, is the result of factors that damage the nasal mucosal lining, affect the vessel walls, or alter the coagulability of the blood, which may be categorized into environmental, local, systemic, and medication-related causes [1]. It is a common disorder in both children and young adults; however, it is uncommon in newborns and children [2]. Epistaxis is a common presentation in the emergency department (ED), with around 345,000 ambulatory care visits in the United States in 2017 [3]. Approximately 60% of people will have at least one episode in their lives, which usually takes the form of a bimodal distribution, with incidence peaks in children under the age of 10 and adults over the age of 35 [4-6]. Although bleeding is frequently self-limiting or stops with external nasal compression, roughly 6% of epistaxis episodes will necessitate invasive intervention to stop the bleeding, accounting for approximately one in every 200 ED visits in the United States [7,8]. It most usually occurs between the ages of three and eight years old, with the prevalence decreasing in maturity [9]. The incidence of epistaxis was observed to be 10%-60% among adults, while 50% of all adult persons had epistaxis as a child [10]. Annually, 7%-14% of the general population was admitted to emergency rooms with epistaxis. Nasal bleeding can be caused by either systemic or local factors, with the former including blood disorders, anticoagulant use, and coagulopathy, while the latter including trauma, septal perforation, nasal allergies, upper airway infections, and the introduction of foreign bodies into the nasal cavity [11]. The emergency therapy for epistaxis is first aid, which is essential to reduce discomfort and avoid the progression of the situation, which can be achieved by pressure or spontaneously [12]. In a study conducted in Saudi Arabia, the prevalence of epistaxis among children was found to be 39.5%, and it was acute in 70.9%. The most common cause of epistaxis was inflammatory diseases (50.5%), and the most common complication reported was recurrent epistaxis (15.2%). Observation alone was the most given management option (69.6%), and recovery was obtained in 100% of patients [13].

School is one of the main locations of pediatric epistaxis because children can have unintentional injuries while playing. School teachers are responsible for the safety and well-being of the students at school. It can be effectively managed at schools with simple measures such as digital compression on the nose and tilting the head forward. It is important for school teachers to be aware of and knowledgeable about the proper first aid procedures in order to deal with epistaxis promptly and appropriately [14-16]. However, it has been shown that teachers, who are typically the first responders for pediatric epistaxis in schools, have an inadequate or poor understanding of and familiarity with appropriate first aid techniques. Evidence suggests that factors such as teachers’ age and educational level, their teaching experience, their previous first aid training, and their exposure to children in need of first aid all affect their first aid knowledge [14]. In a study conducted in the Qassim region of Saudi Arabia, around 20% of participants had a good level of knowledge regarding the first aid management of epistaxis.

The aim of this study is to assess the knowledge and awareness of epistaxis among teachers of different teaching levels from various regions of the Kingdom of Saudi Arabia.

## Materials and methods

Study design

A cross-sectional study was designed to assess the awareness of epistaxis among teachers.

Sample size

The sample size was calculated using the Raosoft sample size calculator by x=Z(c/100)2r(100-r), n=N x/((N-1)E2 + x) and E=Sqrt[(N - n)x/n(N-1)]. The sample size was 439 with a confidence interval of 95%.

Target population

This survey was performed on teachers from different teaching levels including kindergarten, primary, intermediate, and secondary. All of them were Saudi nationals belonging to different regions including Baljurashi, Al Qara, Bani Hassan, Ghamd al Zanad, Al Bahah, Al Mandaq, Qilwah, Al Makhwah Al Hajrah, and Al Aqiq.

Ethical approval

Ethical approval was taken from Imam Mohammad Ibn Saud Islamic University. Initial electronic form consent was also taken from each subject participating in this study. Information provided by each individual was kept completely confidential.

Study tool

A validated and reliable questionnaire was used in this study determined by Cronbach’s alpha. The questionnaire was adapted from a previous study [17] and administered through Google Forms in Arabic language. It consisted of eight demographic questions, which aimed to gather information such as profession, age, gender, nationality, specialty, school type, teaching levels, and work location. These questions were included to provide background information about the participants.

Additionally, the questionnaire included a question regarding whether the participants had received any training on the management of epistaxis (nosebleeds). This question aimed to assess the participants’ previous knowledge and experience in dealing with epistaxis.

Furthermore, the questionnaire comprised two questions related to the participants’ past knowledge of emergency management techniques for epistaxis. Additionally, there were nine questions designed to assess the participants’ awareness and understanding of the methods applied in the management of nosebleeds. The questionnaire was distributed among the relevant subjects.

Statistical analysis

For data analysis, all questions were coded into a spreadsheet and transferred into Statistical Package for the Social Sciences (SPSS) version 23 (IBM SPSS Statistics, Armonk, NY). Data were presented as frequencies and percentages. The association between variables was measured using a chi-square test (p<0.05 was considered significant).

## Results

Demographics

In this cross-sectional study performed on 439 individuals, 49.2% were males and 50.8% were females; all were Saudi nationals. Of the participants, 1.8% were less than 25 years, 16.6% were 25-35 years, 47.6% were 36-45 years, and 33.9% were above 46 years, of which 91.5% of them belonged to academia. More than 95% of the aforementioned academicians were working in government sectors, while only 3.2% were in private institutes. The working area of the majority of the participants (42.1%) was the Al Bahah region, followed by Baljurashi (28.2%), and only 0.5% were working in Al Hajrah. Most of the participants were primary school teachers (30.75%), and 53.9% were from a literature background, while 49.1% were from a scientific background (Table 1).

**Table 1:** Demographic characteristics

Demographic	Frequency	Percentage
Teacher		
Yes	401	91.5%
No	38	8.5%
Total	439	100%
Age group		
25 years or less	8	1.8%
25-35 years	73	16.6%
36-45 years	209	47.6%
46 years or above	149	33.9%
Total	439	100%
Sex		
Male	216	49.2%
Female	223	50.8%
Total	439	100%
Nationality		
Saudi	439	100%
Total	439	100%
Work location		
Baljurashi	124	28.2%
Al Bahah	185	42.1%
Bani Hassan	12	2.7%
Al Aqiq	28	6.4%
Al Mandaq	13	3%
Al Mukhwah	9	2.1%
Al Qara	15	3.4%
Ghamd al Zanad	9	2.1%
Al Hajrah	2	0.5%
Qilwah	4	0.9%
Total	439	100
School type		
Government	425	96.8%
Private	14	3.2%
Total	439	100%
Teaching level		
Kindergarten	4	0.9%
Primary	135	30.75%
Secondary	130	29.6%
Intermediate	94	21.4%
Intermediate, secondary	29	6.6%
Primary, intermediate, secondary	10	2.2%
Primary, intermediate	27	6.1%
Kindergarten, primary, medium, secondary	1	0.2%
Primary, secondary	2	0.4%
Kindergarten, primary	3	0.6%
Kindergarten, intermediate, secondary	2	0.4%
Kindergarten, primary, intermediate,	2	0.4%
Total	439	100%
Specialty		
Literary	237	53.9%
Scientific	202	46.1%
Total	430	100%

Training regarding the management of epistaxis

Out of 439 teachers, 50.7% had received training regarding epistaxis, while 49.3% had never been trained. Table 2 shows the data on the number of individuals who had been trained for the management of epistaxis. No association between age, gender, and working area, and the training received regarding the management of epistaxis was found (p<0.05).

**Table 2:** Training regarding the management of epistaxis

Training received	Frequency	Percentage
Yes	223	50.7%
No	216	49.3%
Total	439	100%

Cognizance regarding epistaxis among teachers

Of the participants, 73.3% considered that applying nasal compression may help stop bleeding, while the rest were completely unaware. On further investigation, 49.8% and 37.1% said that applying pressure on the top of the nose for less than two minutes and 2-5 minutes of compression would be fruitful in epistaxis, respectively. Tilting the head forward would be done by 56.27% of the teachers, while 40.73% said that the head should be tilted backward. Furthermore, 52.6% considered using gauze to stop nasal bleeding, while around 60% reported that putting ice on the head and nose in the case of epistaxis would be helpful. The majority of subjects (81.1%) would use no other way, while many other ways would be used by the rest of the participants for the management of epistaxis. Of the teachers, 53.3% would definitely go to the emergency department if bleeding continued for more than 10 minutes. Teachers from a scientific background as compared to those from a literature background were able to answer better regarding the management of epistaxis (p<0.05). Table 3 shows the abovementioned data.

**Table 3:** Data regarding cognizance of epistaxis

Cognizance of epistaxis among teachers	Frequency	Percentage
Has one of your male/female students or one of the school’s employees ever had a nosebleed?		
Yes	294	66.9%
No	145	33.1%
Total	439	100%
If you encounter a nosebleed, how will you try to stop the bleeding?		
Apply nasal compression	323	73.3%
Let it bleed without pressure	41	9.1%
I don’t know	75	17.5%
Total	439	100%
If you press the nose to stop the bleeding, where will you press it?		
Top of the nose	306	69.7%
Below the nose	133	30.3%
Total	439	100%
How long will you continue to hold pressure on the nose to stop the bleeding?		
Less than 2 minutes	163	37.1%
2-5 minutes	219	49.8%
6-10 minutes	40	9.1%
11-20 minutes	15	3.4%
More than 20 minutes	2	0.5%
Total	439	100%
How will you change the position of the head?		
Head tilted forward	247	56.27%
Head tilted backward	192	40.73%
Total	439	100
Will you block the nose with tissue or gauze?		
Yes	231	52.6%
No	208	47.4%
Total	439	100
Will you put ice on the head or nose?		
Yes	253	57.6%
No	148	33.7%
Missing data	38	8.7%
Total	439	100%
Will you use other ways to stop the bleeding?		
No	356	81.1%
Not to fill the napkins or gauze inside the nose	2	0.45%
Ask for help from a more experienced person	2	0.45%
Wash the head with water	3	0.68%
Request for an ambulance and placement of chatter	2	0.45%
Yes (make it with the vacuum below)	4	0.9%
Go with the student to the health center	2	0.45%
Place ice on the nose bone	2	0.45%
Wash with cold water	2	0.45%
Keep the head tilted forward	2	0.45%
Let the injured sit in the shade	2	0.45%
Use Vaseline, if any	2	0.45%
Transfer the patient to the hospital	2	0.45%
Look for help from a specialist	2	0.45%
Apply vinegar, if any	2	0.45%
Sit calmly	2	0.45%
Spray water on the head from the back	2	0.45%
Go to the emergency hospital	2	0.45%
Transfer to the health center	2	0.45%
Place cold water on the head	2	0.45%
Pour water into the head	2	0.45%
Remove any bundle around the neck	2	0.45%
Try to calm the injured	2	0.45%
Go to the nearest hospital	2	0.45%
Pour a little water on the back of the head while the patient is bent forward	2	0.45%
Use napkin	2	0.45%
Go to the nearest health center	2	0.45%
Use cold water	2	0.45%
Use napkins and replace them if they are full of blood	2	0.45%
Place Vaseline in the nose	2	0.45%
Lay flat on the back	2	0.45%
Use water to wash the middle of the head	2	0.45%
Use the nearest health center	2	0.45%
Contact the ambulance	2	0.45%
Place ice or cold water on the head	2	0.45%
Use ice	2	0.45%
Wash the head with cold water	2	0.45%
Put ice and press the nose	2	0.45%
Water	2	0.45%
Wash it with cold water	2	0.45%
Total	439	100%
When should you go to the emergency department in cases of nose bleeding?		
If the bleeding continues for more than 10 minutes	233	53.1%
If the bleeding continues for more than 30 minutes	130	29.6%
If the bleeding continues for more than 60 minutes	33	7.5%
I don’t know	43	9.79%
Total	439	100%

## Discussion

Epistaxis, also known as nosebleed, is one of the common ear, nose, and throat emergencies present in primary care units that require medical attention. In the United States, 60% of the population has experienced epistaxis, and medical intervention was needed by only 10%. Deviated nasal septum, hypertension, alcohol, non-steroidal anti-inflammatory drugs (NSAIDs), steroidal nasal sprays, and coagulopathies are the major causes of nosebleeds [15].

Our study showed that 49.3% of teachers were aware of and received training for the management of nosebleeds. Similarly, another study showed that 68% of teachers in Riyadh, Saudi Arabia, attended the course for the management of epistaxis. Teachers reported using nose pressure as a way of controlling epistaxis in 76.5% of cases, with 23% mentioning the lower part as the place to press and 12.8% mentioning forcing for 6-10 minutes. When asked about whether the nose should be obstructed, 55.9% reported that it should not be obstructed. Also, 78.3% of the teachers reported the importance of changing head position, while 60.2% selected forward tilted head as the correct position. Putting ice over the nose in case of epistaxis was recorded by 57.4% of the teachers, while 48.4% said that the student should go to the emergency department if bleeding lasts for more than 10 minutes [16]. Another study was performed on the awareness of teachers on epistaxis in the Al-Ahsa region, in which 76% of the teachers were female and 40% were working as primary school teachers. Of the teachers, 54% had received information about first aid to stop nasal bleeding. Furthermore, 15% said that they would not try to stop the bleeding, and only 25% said that they would press on the cartilaginous part of the nose [18]. Data from our cross-sectional study showed nasal compression as a method to stop nasal bleeding was recorded by 73.3% mentioning the upper part as the area to be compressed. The importance of tilting the head forward was reported by 56.27%, and the maximum number of teachers said that using ice in the case of epistaxis is helpful. Of the teachers, 53.1% said that students should refer to the emergency department if bleeding lasts for more than 10 minutes.

In our study, 49.3% of the teachers had received training on the management of epistaxis, while the remaining were untrained. Teachers’ awareness means public awareness, so teachers’ training regarding the management of minor injuries is very important. However, in addition to increasing teachers’ awareness regarding this area through health education programs [16], it is important to notice that teachers should be trained to apply epistaxis management [18].

Limitations

This study has numerous limitations. We cannot establish a causal relationship due to the cross-sectional study design, and our data represent a single time point. Another limitation is related to non-probability sampling for this study as the participants were included through the researchers’ networks and disseminated via social media platforms, questions related to teachers having students with a nosebleed history, and questions about trained teachers provided by what, when, and how long ago. Teachers who were already trained before and those who were not were also compared. Future studies with representative samples are required to strengthen the findings of this study. Further research proposing prevention and management modules or strategies for common causes of epistaxis is highly recommended to reduce its incidence among school-age children.

## Conclusions

Teachers’ training on minor injuries should be a key concern of health educationists as students spend maximum of their time in schools with teachers, and teachers should know what to do as first aid in the management of this type of situation. Our study data showed that the majority of the teachers had insufficient knowledge of first aid to manage minor injuries. Furthermore, in this study, we investigated what methods can be used by teachers to stop nasal bleeding and how many of them were unable to cope with the situation. The lack of awareness of the teachers, which certainly reflects public ignorance on this subject, should be taken seriously.

The study highlights knowledge gaps regarding epistaxis first aid management inside schools in Al-Baha region, Saudi Arabia. The research highlights the need for focused training programs and awareness efforts to enhance teachers’ knowledge and first aid practices. Addressing misknowledge and mispractices, enhancing the practices and attitudes of healthcare providers toward appropriate feeding practices, and promoting a supportive and safe environment could all contribute to the improvement of quality of life and health among the population of Saudi Arabia.

## References

[1] Middleton PM (2004). Epistaxis. Emerg Med Australas.

[2] Varshney S, Saxena RK (2005). Epistaxis: a retrospective clinical study. Indian J Otolaryngol Head Neck Surg.

[3] Pallin DJ, Chng YM, McKay MP, Emond JA, Pelletier AJ, Camargo CA Jr (2005). Epidemiology of epistaxis in US emergency departments, 1992 to 2001. Ann Emerg Med.

[4] Birmingham AR, Mah ND, Ran R, Hansen M (2018). Topical tranexamic acid for the treatment of acute epistaxis in the emergency department. Am J Emerg Med.

[5] Bridwell RE, April MD, Long B (2019). Does oral or topical tranexamic acid control bleeding from epistaxis?. Ann Emerg Med.

[6] Reuben A, Appelboam A, Barton A (2019). Novel use of tranexamic acid to reduce the need for Nasal Packing in Epistaxis (NoPac) randomised controlled trial: research protocol. BMJ Open.

[7] Akkan S, Çorbacıoğlu ŞK, Aytar H, Emektar E, Dağar S, Çevik Y (2019). Evaluating effectiveness of nasal compression with tranexamic acid compared with simple nasal compression and Merocel packing: a randomized controlled trial. Ann Emerg Med.

[8] Zahed R, Moharamzadeh P, Alizadeharasi S, Ghasemi A, Saeedi M (2013). A new and rapid method for epistaxis treatment using injectable form of tranexamic acid topically: a randomized controlled trial. Am J Emerg Med.

[9] Sudarshan PB, Sundaravadanan BS, Prabu Shankar S (2017). A comparative study of totally extraperitoneal versus transabdominal preperitoneal repair of inguinal hernias. Int Surg J.

[10] Davies K, Batra K, Mehanna R, Keogh I (2014). Pediatric epistaxis: epidemiology, management & impact on quality of life. Int J Pediatr Otorhinolaryngol.

[11] Faistauer M, Faistauer A, Grossi RS, Roithmann R (2009). Clinical outcome of patients with epistaxis treated with nasal packing after hospital discharge. Braz J Otorhinolaryngol.

[12] Gupta AK, Jain S, Pal Singh D, Jindal A, Singh K (2009). Epistaxis: management protocol as per etiology. Clin Rhinol.

[13] Alqarni ZM, Alajmi TA, Alhumaidi UH, Alhussain A, Alotaibi YM, Alzahrani HS (2019). Prevalence, causes, treatment, and outcome of epistaxis. IJMDC.

[14] Alanazy S, Alqunibut I, Albahli R (2023). The level of school teachers’ knowledge about first-aid management and control of epistaxis in Qassim Region, Saudi Arabia. Cureus.

[15] Tabassom A, Dahlstrom JJ (2022). Epistaxis. Epistaxis.

[16] Al-Kubaisy Y, Suwayyid WK, Al-Shakhs AA (2019). Teachers’ awareness regarding first-aid management and control of epistaxis inside schools in Riyadh region, Saudi Arabia. Int J Med Dev Countries.

[17] Alasiri AS, Magboul NA, Alasiri AB (2022). Teacher’s awareness regarding epistaxis first-aid management inside schools in Asser Region, Saudi Arabia. Egypt J Otolaryngol.

[18] Alshehri F, Alluwaim F, Alyahya K (2018). Teachers’ awareness regarding emergency management of epistaxis inside the school; Alahssa, Saudi Arabia. Open J Prev Med.

